# Development of an Ultrasensitive and Rapid Fluorescence Polarization Immunoassay for Ochratoxin A in Rice

**DOI:** 10.3390/toxins12110682

**Published:** 2020-10-29

**Authors:** Xiaorong Huang, Xiaoqian Tang, Abdoulie Jallow, Xin Qi, Wen Zhang, Jun Jiang, Hui Li, Qi Zhang, Peiwu Li

**Affiliations:** 1 Oil Crops Research Institute, Chinese Academy of Agricultural Sciences, Wuhan 430062, China; huangxiaorong@caas.cn (X.H.); tangxiaoqian@caas.cn (X.T.); 2019y90200006@caas.cn (A.J.); 2 Key Laboratory of Biology and Genetic Improvement of Oil Crops, Ministry of Agriculture, Wuhan 430062, China; lihui04@caas.cn; 3 Laboratory of Quality & Safety Risk Assessment for Oilseed Products (Wuhan), Ministry of Agriculture, Wuhan 430062, China; 4 Key Laboratory of Detection for Mycotoxins, Ministry of Agriculture, Wuhan 430062, China; 5 Quality Inspection & Test Center for Oilseed Products, Ministry of Agriculture, Wuhan 430062, China; qixin@caas.cn (X.Q.); zhangwen@oilcrops.cn (W.Z.); jiangjun@caas.cn (J.J.)

**Keywords:** FPIA, mycotoxin, OTA, detection methods, food safety, monoclonal antibody (mAb), tracer, HPLC

## Abstract

Ochratoxin A (OTA) is a known food contaminant that affects a wide range of food and agricultural products. The presence of this fungal metabolite in foods poses a threat to human health. Therefore, various detection and quantification methods have been developed to determine its presence in foods. Herein, we describe a rapid and ultrasensitive tracer-based fluorescence polarization immunoassay (FPIA) for the detection of OTA in rice samples. Four fluorescent tracers OTA-fluorescein thiocarbamoyl ethylenediamine (EDF), OTA-fluorescein thiocarbamoyl butane diamine (BDF), OTA-amino-methyl fluorescein (AMF), and OTA-fluorescein thiocarbamoyl hexame (HDF) with fluorescence polarization values (δFP = FPbind-FPfree) of 5, 100, 207, and 80 mP, respectively, were synthesized. The tracer with the highest δFP value (OTA-AMF) was selected and further optimized for the development of an ultrasensitive FPIA with a detection range of 0.03–0.78 ng/mL. A mean recovery of 70.0% to 110.0% was obtained from spiked rice samples with a relative standard deviation of equal to or less than 20%. Good correlations (*r*^2^ = 0.9966) were observed between OTA levels in contaminated rice samples obtained by the FPIA method and high-performance liquid chromatography (HPLC) as a reference method. The rapidity of the method was confirmed by analyzing ten rice samples that were analyzed within 25 min, on average. The sensitivity, accuracy, and rapidity of the method show that it is suitable for screening and quantification of OTA in food samples without the cumbersome pre-analytical steps required in other mycotoxin detection methods.

## 1. Introduction

Ochratoxin A (OTA) is the poisonous secondary metabolite excreted by *Penicillium* and *Aspergillus* species, which is often found in a wide range of foods, such as rice, beans, wine, beer, coffee, cocoa, dried fruit, and animal products. OTA is categorized by the International Agency for Research on Cancer (IARC) as a group 2B possible human carcinogen. It is hepatotoxic, teratogenic, immunosuppressive, nephrotoxic, and nephrocarcinogenic [1,2]. A number of countries have moved to establish regulatory limits on OTA in food products destined for human consumptions [3]. For instance, the European Commission has imposed regulatory limits on OTA in corn and corn products. A maximum of 5 μg/kg for natural corn grain, 3 μg/kg for all other corn products destined for direct human consumption, and 0.5 µg/kg for baby food and corn-based products intended for young children is allowed [4].

To safeguard human health against the food safety risks associated with OTA, advanced, sensitive, and accurate analytical methods are required for its detection and quantification [4]. Instrument-based methods like HPLC connected to a fluorescence detector (HPLC/FLD) and liquid chromatography/mass spectrometry (LC/MS) are some of the most widely-used mycotoxin detection techniques. While instrument-based methods offer precision and reliability, compared to newer analytic techniques, they have some weaknesses: they are costly, require a certain level of expertise to operate them, and are not suitable for on-site use [5,6]. To overcome these drawbacks, immunoassays have recently gained popularity as an alternative to the above-described methods. Based on the binding of antigen to antibody, immunoassay-based techniques are cheap, simple, and sensitive [7,8].

Certain immunoassay techniques, such as ELISA, require tedious and time-consuming assay development [8]. Alternatively, FPIA is a simple and user-friendly immunoassay as it does not require tedious and time-consuming pre-analytical steps [9,10]. FPIA is a widely used homogeneous-based immunoassay with simple and rapid operational procedures. Currently, the method is widely applied in the monitoring of small molecules in variety of matrices [11,12,13]. Fluorescence polarization (FP) is commonly used to excite fluorescent molecules with polarized light in a vertical direction, and then measure the fluorescence intensity Iv and Ip of polarized light emitted in the vertical and horizontal directions, respectively. FP = (IV-IP)/(Iv + Ip), where FP is a dimensionless quantity, and the unit is usually expressed in mP. The principle of fluorescence polarization detection is based on the different sizes of the fluorescence molecules and the different intensities of the fluorescence polarization signal [14].

The use of FPIA to detect mycotoxins such as aflatoxins (AFTs), fumonisins (FBs), deoxynivalenol (DON), ochratoxin A (OTA), zearalenone (ZEN), and HT-2 and T-2 toxins in various matrices as reviewed by Maragos [4]. Additionally, Li et al. reported the development of a multiplexed FPIA for the simultaneous determination of deoxynivalenol, T-2 toxin, and fumonisin in maize samples. With regards to OTA, an OTA-ethylenediamine fluorescence (EDF) conjugate-based FPIA with a limit of detection (LOD) of 0.3 ng/mL of OTA in unpolished rice was reported [15]. In this study, we synthesized four tracers, among which the OTA-AMF tracer was chosen for further optimization to improve the detection sensitivity.

In this work, four new tracers with different fluorophores were synthesized. Based on fluorescence intensity, we selected two apparently better tracers for the subsequent experiments. Two FPIAs were then researched for the determination of OTA in buffer by optimizing the reaction conditions. Based on the optimum tracers and sensitive antibody against OTA, we successfully built and applied a simple, fast, and sensitive FPIA for the detection of OTA in rice. Based on optimal conditions, we further validated the results that were obtained by FPIA using HPLC as a reference method. The developed FPIA, as a result, is a promising method for the rapid analysis of OTA-contaminated rice samples.

## 2. Results and Discussions

### 2.1. The Principle of FPIA

The principle of this type of immunoassays is based on the competition between native mycotoxins in the sample and the mycotoxin-labeled tracer for the monoclonal antibody (mAb) [16]. The addition of the tracer to the mAb influences the tracer molecule activation and enhances the FP value [17]. The quantity of synthetic tracer is inversely proportional to the amount of free mycotoxin that exists in the sample; consequently, the analyte concentration inversely correlates with the polarization value. Specifically, the small molecule to be measured is labeled with a fluorescent substance capable of generating polarized light and the change of FP value before and after the fluorescence marker is combined with specific antibody is measured (δFP = FPbind-FPfree). Then, a standard curve is established to achieve quantitative detection of the small molecule to be measured.

### 2.2. Preparation of Monoclonal Antibody

**Figure 1:**
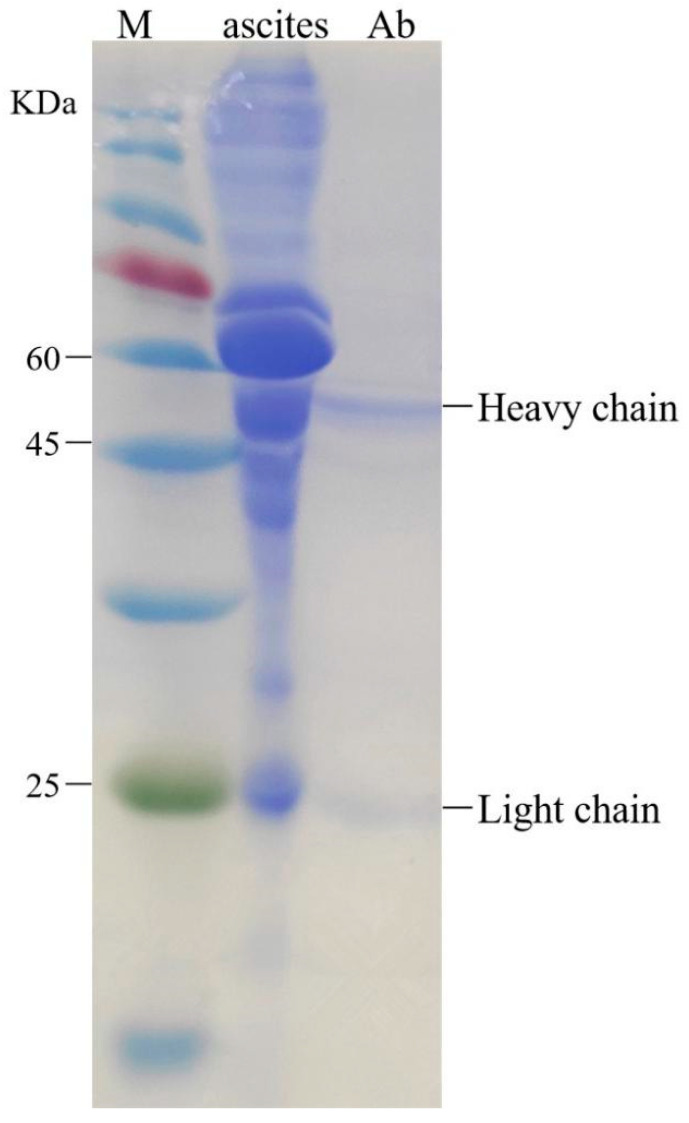
Purification and identification of antibody by SDS-PAGE electrophoretogram.

The 1H2 cells reached logarithmic phase four days after resuscitation. Ascites were collected 10 days after injection. The purification effect of the monoclonal antibody was determined with SDS-PAGE (12% separation gel, 5% spacer gel). The purified immunoglobulin G antibody has only two main bands, heavy chain and light chain, indicating that the purification method can remove the heteroprotein in ascites. The relationship between the molecular weight of the protein marker and the mobility of the heavy and light chains was obtained. The molecular weights of heavy and light chains were about 50 and 25 kDa, respectively (Figure 1). The purified antibodies proved to be capable of meeting the requirements for the next experiment.

### 2.3. Synthesis of the OTA-FL Tracer

The carboxyl groups in OTA are inactive; for their activation, we used N, N’-dicyclohexylcarbodiimide and N-hydroxysuccinimide in an aprotic solvent medium. In this research, four typical kinds of dyes with different Ex/Em wavelengths (HDF, BDF, AMF, and EDF) were selected for covalent conjugation to OTA.

**Figure 2:**
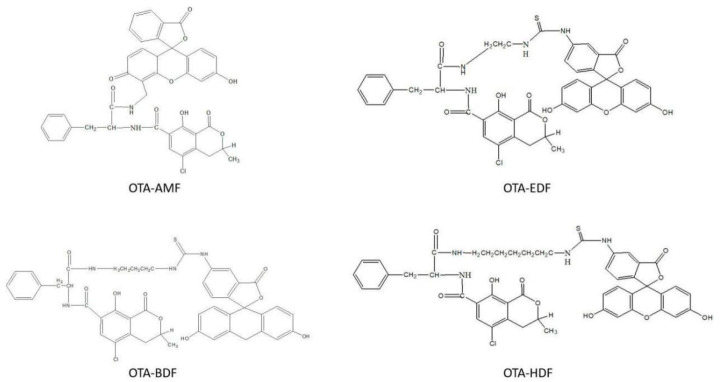
Structural formulas of tracer OTA-AMF, OTA-BDF, OTA-EDF, and OTA-HDF.

**Figure 3:**
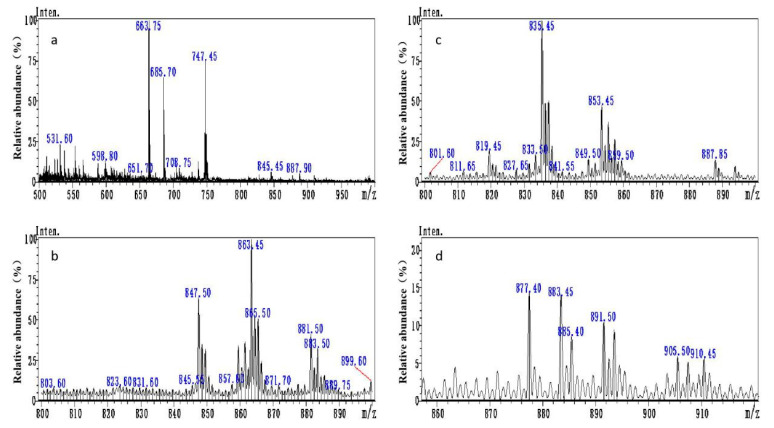
Mass spectra of (**a**) OTA-AMF, (**b**) OTA-BDF, (**c**) OTA-EDF, and (**d**) OTA-HDF.

**Figure 4:**
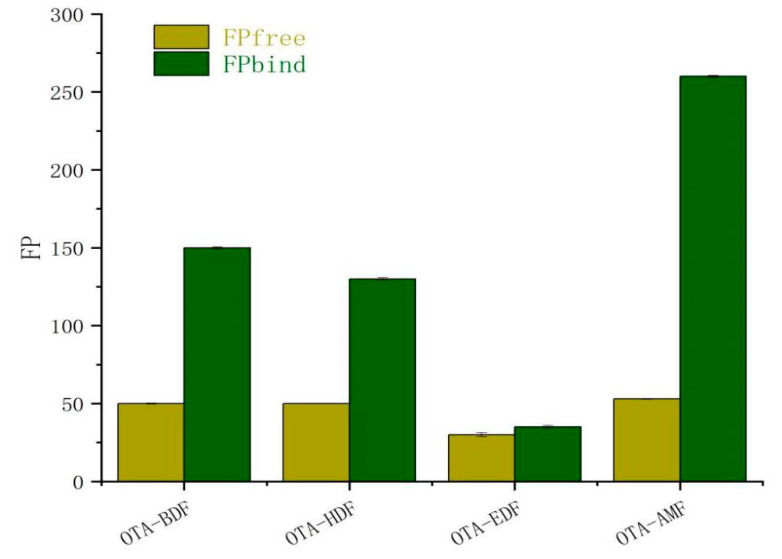
The result of four tracers combined with the diluted specific monoclonal antibodies (*n* = 3).

After preliminary purification through thin layer chromatography (TLC), principal bands of OTA-AMF, OTA-BDF, OTA-EDF, and OTA-HDF were collected. The molecular weights of OTA-AMF, OTA-BDF, OTA-EDF, and OTA-HDF were 747.15, 863.33, 837.29, and 891.38, respectively (Figure 2). The mass spectra [M+] ion peaks were 747.45, 863.45, 835.45, and 891.50, respectively, which are consistent with the molecular weights of the target compounds (Figure 3). Dye-labelled tracers were primarily designed to bind to the specific monoclonal antibodies to determine whether the OTA-AMF, OTA-BDF, OTA-EDF, and OTA-HDF could provide satisfactory results. All the tracers induced a significant rise in FP signals before and after the addition of saturated quantities of mAbs (Figure 4). The δFP values of the tracers ranged from 5 to 207 mP, which are adequate for application in FPIA reaction progress monitoring. This proved that the dyes were successfully conjugated to the corresponding mycotoxin [18]. To improve the detection sensitivity, the OTA-AMF that had the highest δFP value was chosen for further optimization.

### 2.4. Optimization of the FPIA

**Figure 5:**
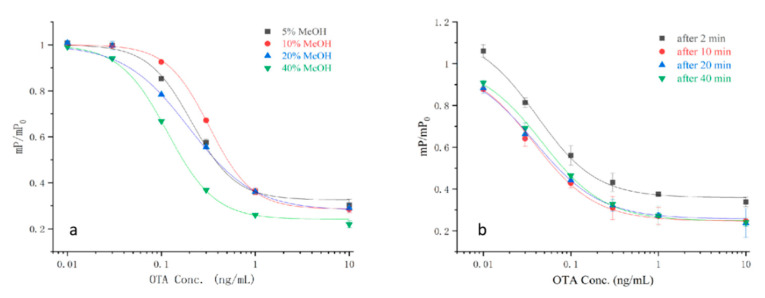
Normalized correction curves of the optimum FPIA implemented with OTA reference solutions with different methanol content (**a**) and reaction time (**b**) (*n* = 3).

The influence of methanol concentration and reaction time on the assay’s performance was studied to evaluate the applicability of the method. OTA is typically extracted from cereals with methanol. The dyes that were applied in this experiment were methanol-sensitive substances; various physicochemical statuses can importantly affect the cross-linkage between the antigens and antibodies [19,20]. As the methanol concentration rose from 0% to 40%, the half maximal inhibitory concentration (IC_50_) of the experiment slightly decreased (Figure 5a). The reaction time was studied at a concentration of 40% methanol. The best level of sensitivity was attained at 0–10 min; the IC_50_ values increased with the increase of the assay time (Figure 5b). Therefore, 40% methanol and 20 min reaction time were regarded as the optimum assay conditions and were applied in the subsequent experiments.

**Figure 6:**
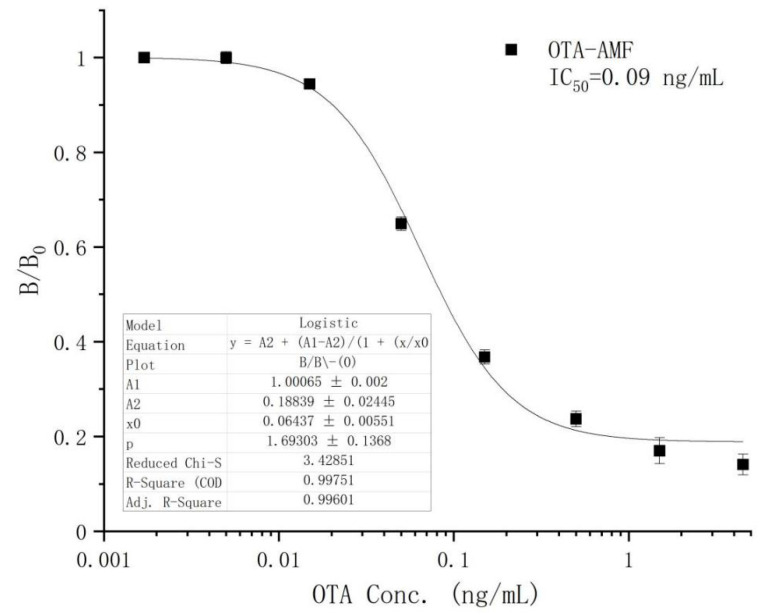
The standard curve of OTA.

Based on the optimum conditions, we established the OTA calibration curve within the concentration range of 0.03–0.78 ng/mL. When the correlation coefficient was equivalent to 0.996, the limit of detection (LOD) and IC_50_ were 0.02 and 0.09 ng/mL, respectively (Figure 6). Due to the dilution ratio of rice being 20:1, the linear detection range of this method for rice in the actual detection was 0.60–15.60 ng/mL.

### 2.5. Evaluation of FPIA

The recovery, intra-assay, and inter-assay tests were carried out to assess the performance of the FPIA method. The recovery tests in rice samples spiked with OTA standards of 0.5, 5, and 50 μg/kg concentration (Table 1) yielded an average recovery range of 70–110% with a relative standard deviation of equal to or less than 20%. The recovery and reproducibility values of this method proved that it fits the mycotoxin screening and testing methods’ validation and performance criteria standard set by the EU for the control of mycotoxin in food products [4].

### 2.6. FPIA Screening and HPLC Analysis of Blind Samples

**Figure 7:**
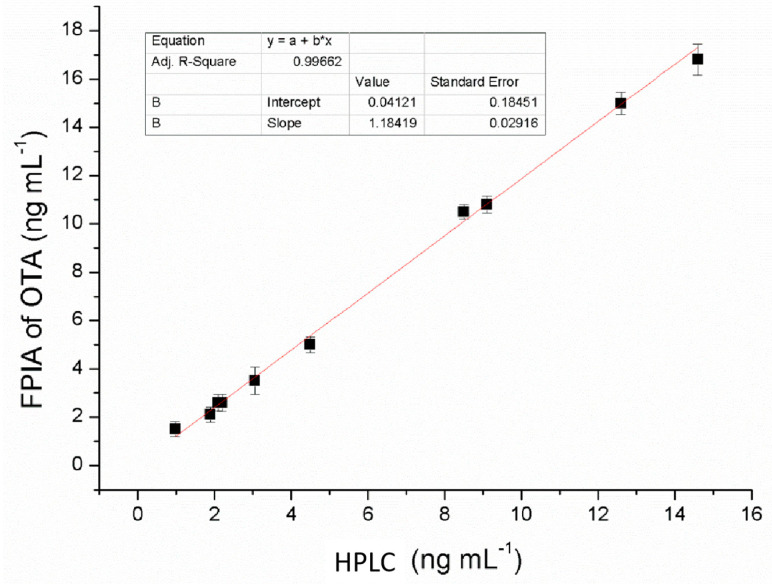
Correlation analysis between HPLC method and the developed FPIA assy.

A set of 10 naturally OTA-contaminated rice samples with a contamination levels ranging from 0.98 to 14.6 ng/mL (through HPLC analysis) was analyzed by both an immunoaffinity column clean-up with HPLC and the developed FPIA assay for comparison (Table 2). Linear regression of recovery data showed a good correlation (*r*^2^ = 0.9966, Figure 7). An analysis of variance (ANOVA) estimation proved that OTA concentrations results obtained through the FPIA method were a good forecast of the expected values as tested by HPLC (*p* < 0.0001). Data from the two methods showed a good level of correlation and concurred with the spiked quantities, proving the practical applicability of the developed method.

## 3. Conclusions

A sensitive and accurate FPIA analytical method based on a new tracer (OTA-AMF) was developed and optimized for the rapid detection and quantification of OTA in rice and maize samples. The method detected OTA in concentrations ranging from 0.03–0.78 ng/mL and reached an IC_50_ value of 0.09 ng/mL. Compared to other immunoassays (Table 3), the FPIA method has better sensitivity. Apart from the purification procedure, the whole analytical process was completed within 10 min showing its practical applicability as a rapid screening method. The precision of the method was proven by comparing the results of OTA levels in rice and maize samples obtained by HPLC and results obtained by the FPIA. Furthermore, the proposed technique is inexpensive, user-friendly, and suitable for on-site use. The method is suitable for screening and quantitative determination of OTA in rice and maize at levels lower the EU regulatory limits for OTA in cereal grains and can serve as an alternative to instrument-based methods.

## 4. Materials and Methods 

### 4.1. Reagents and Chemicals

Ochratoxin A (OTA), N,N-dimethylformamide (DMF), 1-ethyl-3-(3-dimethylaminopropyl) carbodiimide (EDC), N-hydroxysuccinimide (NHS), octanoic acid, ammonium sulfate, dimethyl sulfoxide (DMSO), and Freund’s incomplete adjuvant liquid (FICA) were bought from Sigma-Aldrich. Modified RPMI medium, HEPES, and penicillin-streptomycin solution were obtained from GE Healthcare-Hyclone. Fetal bovine serum was obtained from Gibco. Cell culture flask (75 cm^2^) was obtained from CORNING. Dialysis tubing (MWCO 15 KDa) was obtained from Thermo-Scientific. The SDS-PAGE gel preparation kit was obtained from Biosharp Life Sciences. Thin-layer chromatography (TLC) plates were obtained from Darmstadt; the model specifications were silica gel 60, 1 mm, 20 × 20 cm, with fluorescent indicator. Fluorescein thiocarbamoyl ethylenediamine (EDF), fluorescein thiocarbamoyl butane diamine (BDF), fluorescein thiocarbamoyl hexame (HDF), and amino-methyl fluorescein (AMF) were provided by Sergei A. Eremin, a professor of Department of Chemistry, Lomonosov Moscow State University. All FPIA experiments used sodium borate solution (BB, 0.05 M, pH = 9.0). Phosphate buffer solution (PBS, 0.01 M, pH = 7.4) was used for the mAb dialysis. All of the organic solvents and chemicals were reagent grade or above.

The fluorescence polarizer was a sentinel 200 FP portable unit (Diachemix, Grayslake, 1 L). Measurements of intensity and fluorescence polarization were executed applying the TDx/FLx Analyzer (Abbott, Irving, TX, USA) in an aided Photo Check method. TDx/FLx glass cuvettes were put into the particular turntable up to 10 at a time, then polarization (mP units) and fluorescence intensity (customary units) were measured. The total measurement time of 10 samples was about 7 min.

### 4.2. Animals and Cells

Female BALB/c mice(age 8–10 weeks), which were used for the production of antibodies, were purchased from the Institute of Biological Products of Hubei Province (Wuhan, China). OTA hybridoma cell strain 1H2 was developed in our laboratory [37].

### 4.3. Hybridoma Cell Culture and Antibody Preparation

OTA hybridoma cell strain 1H2 was taken out of a liquid nitrogen container, melted in a 37 °C water bath for 1 min, and washed with modified RPMI medium solution. The cells were resuspended with 1640 complete medium (RPMI medium modified: HEPES: penicillin-streptomycin, *V*:*V*:*V*:*V* = 80:20:1:1), and transferred to a cell culture flask, then incubated in a constant temperature incubator (at 37 °C, CO_2_ 5%). Hybridoma cells were collected during the logarithmic phase, and injected into the BALB/c mice that had been treated with FICA. The number of cell injections was maintained at 2 × 10^6^. Ascites were harvested with drainage needle about one week later.

The ascites were used to prepare pure OTA antibodies, which were purified by caprylic acid-ammonium sulfate precipitation [38]. Subsequently, the antibodies were freeze-dried in a vacuum, and stored at −20 °C for later use. The purity of monoclonal antibody was determined by the SDS-PAGE gel electrophoresis method.

### 4.4. Preparation of Four Different OTA-Fluorescein Tracers

OTA has an active group that can be coupled with commonly used fluorescein and directly reacted with FITC to become a tracer OTA-EDF (or OTA-BDF, OTA-AMF, and OTA-HDF) as previously reported [39,40]. In brief, 250 μL (80 μmol/mL) NHS and 250 μL (80 μmol/mL) EDC were dissolved in 0.2 mL DMF, then 2 mg (5 μmol) OTA was added. The solution was stirred for 2 h and subsequently reposed overnight at room temperature. For the synthesis of OTA-EDF, 2 mg (4 μmol) EDF was added into 200 μL (2 μmol) activated OTA solution, followed by 10 h incubation at room temperature. For OTA-BDF and OTA-HDF, 1 mg (2 μmol) BDF (or HDF) was added into 100 μL (1 μmol) activated OTA solution, followed by 10 h incubation at room temperature and an aliquot. For OTA-AMF, 0.5 mg (1 μmol) AMF was added into 100 μL (1 μmol) activated OTA solution after 10 h reaction at room temperature. 

An aliquot of the mixture was separated and purified by TLC. The silica gel plates were activated at 110 °C for 30 min before use. Toluene ± acetic acid (99:1 *v*/*v*) was chosen as the spotting solvent. Toluene ± ethyl acetate ± 88% formic acid (6:3:1 *v*/*v*/*v*) was regarded as the suitable solvent for eluting samples on TLC plates. The TLC plates were examined visually under UV light at 365 nm [36].

The OTA-AMF and other tracers were dissolved in 2 mL and 1 mL methanol, respectively, for separation and purification. A primary band at Rf¼ 0.9 and Rf¼ 0.7 were collected and eluted with methanol. The methanol tracer solutions were stored at −20 °C.

Dye-labeled tracers were identified by mass spectrometry (SHIMADZU, LCMS-8060, liquid chromatograph-mass spectrometer). The instrument was corrected with raffinose before the experiment. The OTA-AMF, OTA-BDF, OTA-EDF, and OTA-HDF were diluted to 1 μg/mL with 1 mL methanol, and then injected into the mass spectrometer through autosampler. The parameters of the mass spectrum were adjusted to real-time at the beginning of data collection. The parameters of the compound ion mass spectrum were obtained and analyzed for their chemical structures.

### 4.5. Method of Fluorescence Polarization Immunoassay

The concentrations of OTA standard working solution were 0, 0.0034, 0.01, 0.03, 0.1, 0.3, 1, 3, and 9 ng/mL prepared with 10% methanol in deionized water. The FPIA was prepared by 0.1 M borate buffer (pH = 7.4); the antibody working fluid was based on attenuating OTA specific antibody (mAb) 1:36,000 in BB buffer. Glass culture tubes with specifications 10 × 75 mm (VWR Scientific) were used as test cuvettes. We added 500 µL antibody working solution into the tube, then 500 µL OTA-EDF (or OTA-BDF/OTA-AMF/OTA-HDF) working solution and mixed. The FP value was measured after 10 min of oscillate incubation at ambient temperature; the relative FP mean values (mP/mP_0_) were used in the inhibition curve to standardize the FP value, where mP is the current FP value of different OTA concentrations and mP_0_ is the value of blank-control (50 µL methanol-BB solution was used as the blank-control) [41]. The values of mP/mP_0_ were plotted against OTA concentration [37]. For experiments to elucidate the reaction’s kinetics, measurements were recorded for time intervals ranging from 3 s to 10 min at room temperature, unless otherwise noted. OTA content of naturally contaminated rice samples was approximated based on the OTA-PBS solution specification curve [42].

### 4.6. Sample Preparation

A total of 5 g of rice was extracted and vortex mixed with 25 mL 50% methanol for 30 min. Then, the supernatant fluid was first filtered with a double filter paper before it was further purified with a 0.22 μm filter membrane. Then, 250 μL of the filtrate was watered down with 750 μL deionized water, samples of which were analyzed with the FPIA method [43].

### 4.7. Comparison with HPLC Analysis

Samples were extracted with ochratoxin A immunoaffinity columns similar to those previously described. Briefly, rice samples were finely ground and homogenized and then mixed with 80% acetonitrile and half volume hexane by gently mixing. The extracts were filtered with a filter paper, and then the filtrate was collected and centrifuged. The bottom layer was evaporated to dry below a flow of nitrogen. After dilution with acetonitrile and PBS, the extracted samples were loaded into the IAC columns. OTA was eluted with 2% methanol/acetic acid solution, and then dry-evaporated. A 50 μL reconstituted sample (1 mL acetonitrile) was injected into the chromatograph [44,45]. The HPLC analysis was run applying a C18 column on a Waters Alliance 2695 chromatographic system in isocratic conditions at ambient temperature with the moving phase of CH_3_CN:NH_3_/NH_4_Cl (20 mM, pH = 9.8) (*v/v* = 15:85); the column was acquired using Waters XTerra^®^ (3 μm, 2.1 × 250 mm), the injection volume was 20 μL, and the flow velocity was 0.2 mL per minute; the FLD determination was acquired using a Waters 474 Scanning Fluorescence Detector (λex 380 nm, λem 440 nm; attenuation 32; gain 7 × 100; bandwidth 40 nm); the analyte holding time was 20 times the retention time, corresponding to the column void volume; no chemical compound could be used as an internal reference for the OTA extraction [46].

## Figures and Tables

**Table 1:** Relative standard deviations and average recoveries of OTA from spiked rice samples obtained by FPIA.

Assay	Spiking Level (μg/kg)	Recovery (%)	CV (%)
Intra-assay	0.5	110	5.6
	5	72.5	6.2
	50	80.1	8.5
Inter-assay	0.5	113.2	6.2
	5	76.8	8.4
	50	80.4	9.8

**Table 2:** Quantitative determination of OTA in practical samples with FPIA and HPLC methods.

	Ochratoxin A Determined (ng g^–1^)	
Sample No.	FPIA	HPLC
1	15.1 ± 0.50	14.6 ± 0.31
2	1.5 ± 0.02	0.98 ± 0.05
3	2.1 ± 0.03	1.9 ± 0.04
4	10.5 ± 0.31	8.5 ± 0.28
5	2.6 ± 0.15	2.2 ± 0.10
6	5.0 ± 0.13	4.5 ± 0.12
7	3.5 ± 0.06	3.06 ± 0.03
8	15.0 ± 0.51	12.6 ± 0.27
9	10.8 ± 0.62	9.1 ± 0.16
10	2.6 ± 0.04	2.1 ± 0.06

**Table 3:** Comparing the sensitivity with other immuno-assay methods.

Author	Year	Antibody	Experiment Method	Sample	Sensitivity (IC_50_, ng/mL)	LOD (ng/mL)
This paper	2020	mAb	FPIA	rice	0.09	0.02
Becheva [21]	2020	F(ab’)2	FIA	milk	a	0.08
Beloglazova [22]	2020	mAb	Flow-through Immunoassay	feed	10	
Wang [23]	2020	Nb	FIA	food	0.46	0.12
Chen [7]	2019	mAb	FPIA	yoghurt	9.32	0.82
Zhang [24]	2019	Nb	ELISA	cereals	97	_
Rehmat [25]	2019	mAb	SPR immunoassay	coffee		3.8
Qin [26]	2019	mAb	ELISA	nutmeg	0.146	0.031
Machado [27]	2018	mAb	capillary micro- fluidic immunoassay	feed		40
Tang [28]	2018	Nb	one-step immunoassay	cereal	5	
Soares [29]	2018	mAb	FIA			1
Sun [30]	2018	Nb	ELISA	rice	0.57	0.059
Liu [31]	2017	Nb	ELISA	cereal	0.64	
Lippolis [32]	2017	mAb	FPIA	rye		0.6
Majdinasab [33]	2015	mAb	TRFICA	agro-product		1
Lippolis [4]	2014	mAb	FPIA	wheat	0.48	0.8
Li [34]	2013	mAb	immunochromatographic assay	agro-food		0.5
Bondarenko [35]	2012	mAb	FPIA	grain		10
Zezza [36]	2009	mAb	FPIA	red wine		0.7

^a^ The data were not detected or shown in the paper. mAb: monoclonal antibody. Nb: nanobody. FIA: fluoroimmunoassay. ELISA: enzyme-linked immunosorbent assay. TRFICA: time-resolved fluorescent immunochromatographic assay.
